# A Genome-Scale Metabolic Reconstruction of *Mycoplasma genitalium*, *i*PS189

**DOI:** 10.1371/journal.pcbi.1000285

**Published:** 2009-02-13

**Authors:** Patrick F. Suthers, Madhukar S. Dasika, Vinay Satish Kumar, Gennady Denisov, John I. Glass, Costas D. Maranas

**Affiliations:** 1 Department of Chemical Engineering, The Pennsylvania State University, University Park, Pennsylvania, United States of America; 2 Department of Industrial Engineering, The Pennsylvania State University, University Park, Pennsylvania, United States of America; 3 J. Craig Venter Institute, Rockville, Maryland, United States of America; University of Washington, United States of America

## Abstract

With a genome size of ∼580 kb and approximately 480 protein coding regions, *Mycoplasma genitalium* is one of the smallest known self-replicating organisms and, additionally, has extremely fastidious nutrient requirements. The reduced genomic content of *M. genitalium* has led researchers to suggest that the molecular assembly contained in this organism may be a close approximation to the minimal set of genes required for bacterial growth. Here, we introduce a systematic approach for the construction and curation of a genome-scale *in silico* metabolic model for *M. genitalium*. Key challenges included estimation of biomass composition, handling of enzymes with broad specificities, and the lack of a defined medium. Computational tools were subsequently employed to identify and resolve connectivity gaps in the model as well as growth prediction inconsistencies with gene essentiality experimental data. The curated model, *M. genitalium i*PS189 (262 reactions, 274 metabolites), is 87% accurate in recapitulating *in vivo* gene essentiality results for *M. genitalium*. Approaches and tools described herein provide a roadmap for the automated construction of *in silico* metabolic models of other organisms.

## Author Summary

There is growing interest in elucidating the minimal number of genes needed for life. This challenge is important not just for fundamental but also practical considerations arising from the need to design microorganisms exquisitely tuned for particular applications. The genome of the pathogen *Mycoplasma genitalium* is believed to be a close approximation to the minimal set of genes required for bacterial growth. In this paper, we constructed a genome-scale metabolic model of *M. genitalium* that mathematically describes a unified characterization of its biochemical capabilities. The model accounts for 189 of the 482 genes listed in the latest genome annotation. We used computational tools during the process to bridge network gaps in the model and restore consistency with experimental data that determined which gene deletions led to cell death (i.e., are essential). We achieved 87% correct model predictions for essential genes and 89% for non-essential genes. We subsequently used the metabolic model to determine components that must be part of the growth medium. The approaches and tools described here provide a roadmap for the automated metabolic reconstruction of other organisms. This task is becoming increasingly critical as genome sequencing for new organisms is proceeding at an ever-accelerating pace.

## Introduction

**Figure 1:**
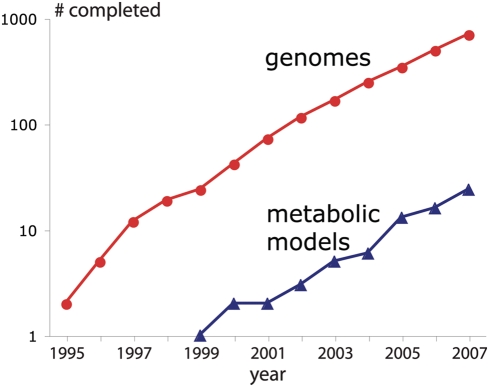
Gap between sequenced genomes and generated models. The numbers of both metabolic models and completed genome sequences have been growing exponentially. Despite the increase in the number of metabolic models, their total remains smaller than 1% of that of sequenced genomes.

Genome-scale metabolic reconstructions are already in place or under development for a growing number of organisms including eukaryotic, prokaryotic and archaeal species [1]. Metabolic pathway reconstructions are increasingly being queried by systems engineering approaches to refine the quality of the resulting metabolic models [2]. Curated metabolic models are indispensable for computationally driving engineering interventions in microbial strains for targeted overproductions [3]–[6], elucidating the organizing principles of metabolism [7]–[10] and even pinpointing drug targets [11],[12]. Currently, over 700 genomes have been fully sequenced [13] whereas only about 20 organism-specific genome-scale metabolic models have been constructed [1],[14],[15]. Figure 1 pictorially demonstrates, in logarithmic space, the widening gap between organism-specific metabolic models and fully sequenced genomes over the past twelve years. It appears that metabolic model generation can only keep pace with about 1% of the fully sequenced genomes.

**Figure 2:**
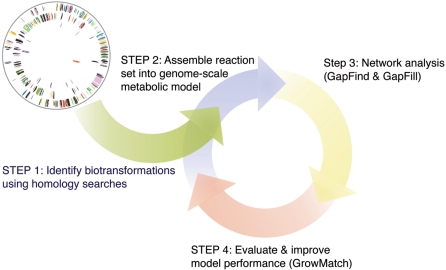
Steps used in the (semi)-automated reconstruction process. The four steps used in the present work are 1) identification of biotransformations using automated homology searches 2) assembly of reaction sets into a genome-scale metabolic model 3) automated network connectivity analysis and restoration, and 4) automated evaluation and improvement of model performance when compared to *in vivo* gene essentiality data.

In response to this flood of present and future genomic information, automated tools such as Pathway Tools [16] and SimPheny (Genomatica) have been developed that, using homology comparisons, allow for the automated generation of draft organism-specific metabolic reconstructions that can subsequently be upgraded into metabolic models. All of these models remain to some extent incomplete as manifested by the presence of unreachable metabolites [17] and some growth inconsistencies between model predictions and observed *in vivo* behavior [2]. In particular, optimization-based techniques for automatically identifying metabolites disconnected from the rest of metabolism (i.e., GapFind) and hypotheses generators (i.e., GapFill) for reconnecting them have recently been introduced [17]. In order to resolve substrate utilization prediction inconsistencies, Reed et al. [2] introduced a novel approach for identifying what reactions to add to the genome-scale metabolic models of *E. coli* to correct some of the *in silico* growth predictions. In our group, we have taken the next step for gene deletion data by attempting to correct all such growth inconsistencies by allowing not just additions but also eliminations of functionalities in the model (i.e., GrowMatch) (Satish Kumar and Maranas, submitted). As outlined in Figure 2, in this work, we describe the application of these automated methodologies during the *Mycoplasma genitalium* model construction process (as opposed to an *a posteriori* mode of deployment).

*M. genitalium* has received considerable attention as it is the smallest organism that can be grown in pure culture, having a genome size of ∼580 kb and approximately 480 protein coding regions [18],[19]. An examination of its genome content revealed limited metabolic capabilities [20], leading researchers to suggest it may be a close approximation to the minimal set of genes required for bacterial growth [19],[21]. Several researchers have carried out genomic and proteomic analysis of *M. genitalium* to quantify this minimal set. For example, Mushegian and Koonin have carried out a detailed comparison of *M. genitalium* and *H. influenzae* proteins to derive a set of 256 genes that they suggested are necessary for viability [22]. Further, genomic analyses of these species revealed that Mycoplasma genes encode for several catabolic and metabolite transport proteins but for only a limited number of anabolic proteins suggesting that Mycoplasma species need to scavenge for the required nutrients from the surrounding environment [20]. More recently, Glass and co-workers performed global transposon mutagenesis and established that 382 of the 482 protein coding sequences are essential genes for this minimal bacterium [19]. These gene sets and essential gene analyses, however, have not been put into context of a complete functional metabolic model.

*Mycoplasma genitalium* is not only the closest known approximation of a minimal cell but also an important sexually transmitted human pathogen. It is a cause of nongonococcal urethritis in men and is associated with genital tract inflammatory diseases in women, including endometritis, cervicitis, pelvic inflammatory disease, and tubal factor infertility (for a recent review see [23]). Additionally, evidence suggests that *M. genitalium* infection increases the risk of contracting HIV-1 [24]–[26]. Mycoplasmas, the generic name for the bacteria that comprise the *Mollicutes* taxon, evolved from the low G+C Gram positive bacteria through a process of massive genome reduction [27]. Their salient characteristics in addition to small genomes are a lack of a cell wall, and an almost complete inability to synthesize the building blocks of DNA, RNA, proteins, and cell membranes.

The above underlines the importance of investigating the molecular biology of mycoplasma and *M. genitalium* in particular. However, a major hindrance to *M. genitalium* research and laboratory diagnosis of infection has been their cultivation *in vitro*. While defined media are present for some mycoplasmas [28]–[30], researchers have often had to resort to complex media to cultivate most mycoplasmas, including *M. genitalium*. *M. genitalium*, and many other mycoplasmas are cultured *in vitro* in SP-4 medium. This extremely rich medium contains several undefined additives including peptones, yeast hydrolysate, yeast extract and 17% fetal bovine serum [31]. The use of complex undefined growth media has interfered with the molecular definition of mycoplasma metabolic pathways, genetic analyses, estimation of growth requirements, characterization of auxotrophic mutants and examining the nutritional control of bacterial pathogenecity.

In this paper, we highlight the development of an *in silico* model of metabolism of *M. genitalium*. It was subjected to network connectivity gap detection and reconnection as well as restoration of consistency with *in vivo* gene essentiality experiments [19]. We subsequently used the model to pinpoint components in the growth medium that are needed for the production of all components of biomass in an effort to eventually eliminate the need for non-defined components such as serum in the growth medium.

## Results

The metabolic model reconstruction process is subdivided into four steps. These four steps are summarized in Figure 2 and include (1) identification of biotransformations using homology searches, (2) assembly of reaction sets into a genome-scale metabolic model, (3) network connectivity analysis and restoration, and (4) evaluation and improvement of model performance when compared to *in vivo* gene essentiality data. Model construction is followed by minimal defined medium component elucidation.

### Reconstruction Content

The metabolic reconstruction of *M. genitalium* was carried out in a series of successive refinements (see Figure 2 and Materials and Methods). Of the 482 predicted open reading frames (ORFs), 113 (23%) only have annotations of (conserved) putative or hypothetical proteins. Of the remainder, 369 ORFs have well-defined annotations, with functions either shown biochemically or predicted for 272 of them (42%) [18],[19]. From these well-annotated genes, 82 (17%) are not involved in specific metabolic transformations, but rather encode proteins whose roles include DNA/RNA polymerization, DNA repair, protein folding and adhesion. The model construction process started with the application of an automated procedure for creating a draft metabolic reconstruction from the genome sequence of *M. genitalium* (see Materials and Methods) [32],[33]. This auto-generated model contained 150 genes and 249 unique metabolites associated with 167 reactions (see Table 1). The 150 genes comprise 31% of the ORFs present in the genome and provided a solid starting point with very little manual effort.

**Table 1:** Composition of the model after each step.

	Automodel^c^	Initial^e^	After GapFill	After GrowMatch (*i*PS189)
Included genes	150 (31%)^d^	187 (39%)	193 (40%)	189 (39%)
Proteins	110	101	105	104
Protein complexes^a^	3	18	19	18
Isozymes	25	2	2	0
Reactions	167	179	265	262
Metabolic reactions	127	138	181	178
Transport reactions	40	41	84	84
Gene-protein-reaction associations				
Gene associated (metabolic/transport)	167	163	170	168
Spontaneous	0	6	6	6
No gene associated (metabolic/transport)	0	10	89	88
Exchange reactions	40	42	89	87
Metabolites^b^	249	263	275	274
Cytoplasmic	234	250	262	261
Extracellular	40	42	85	85

a All ribosome-associated proteins are counted as a single complex. The number for protein complexes and isozymes is not on a reaction basis but rather a protein basis.

b Distinct species ignoring the compartment of the metabolites.

c Includes minor adjustments to compartment assignment.

d Based on 482 protein-encoding ORFs.

e After additional BLASTP matches, specificity adjustments and conversion to computations-ready form.

Additional homology searches of genes not included in the auto-model against the NCBI database increased the number of model components to 187 genes and 263 distinct metabolites associated with 179 reactions (Table 1). These genes comprise 39% of the ORFs present in the genome. These reactions enable, for example, the uptake of glycerol into the cell, thymidine kinase, and ribonucleotide diphosphate reductase (Table 2), as well as the remaining annotated ribosomal proteins that were not previously incorporated. As indicated in Table 2, the bidirectional protein-protein BLAST (i.e., BLASTp) expectation values exhibited by these genes when compared to the biochemically-characterized counterpart in other organisms provided strong support for their inclusion in the model. In addition, we also included nine nucleoside di- and tri-phosphate kinase associated reactions based on the observation that the kinase pool of *M. genitalium* has relaxed substrate specificity [34]. As part of the initial model generation, we also checked the BLASTp scores, gene annotations, and the cluster of orthologous groups (COGs) ontology [35] of all genes in the automodel, to guard against the erroneous inclusion of functions in the model (see Materials and Methods).

**Table 2:** Additional genes and associated reactions added to the model through bi-directional BLASTp homology searches.

Reaction Name	Reaction Abb.	*M. genitalium* Locus	Organism	Locus	Forward E Value	Reverse E Value	Ref.
*Added during initial BLASTP searches*							
glycerol transport	GLYCt	MG033	*B. subtilis*	BSU09280	1E-23	2E-32	[74]
thymidine kinase	TMDK1	MG034	*B. subtilis*	BSU37060	3E-49	1E-46	[75]
inorganic diphosphatase	PPA	MG351	*H. pylori*	HP_0620	3E-29	8E-31	[76]
UTP-glucose-1-phosphate uridylyltransferase	GALU	MG453	*B. subtilis*	BSU35670	2E-69	2E-70	[77],[78]
arginyl-tRNA synthetase	ARGTRS	MG378	*E. coli*	b1876	2E-09	5E-11	[79]
methionyl-tRNA formyltransferase	FMETTRS	MG365	*B. subtilis*	BSU15730	2E-34	1E-40	[80]
phosphotransferase systems^a^	various	MG041	*E. coli*	b2415	1E-07	2E-12	
*Added by GapFill procedure (biomass)*							
glutamyl-tRNA(Gln) amidotransferase	GLXAT	MG098	*M. gallisepticum*	MGA_0414	1E-60	1E-66	
	GLXAT	MG099	*B. subtilis*	BSU06680	3E-50	1E-57	
	GLXAT	MG100	*B. subtilis*	BSU06690	2E-75	3E-76	
NAD kinase	NADK	MG128	*B. subtilis*	BSU11610	9E-16	2E-16	
nicotinic acid mononucleotide pyrophosphorylase	NAMNPP	MG037	*B. subtilis*	BSU31750	5E-05	8E-06	
*Added by GapFill procedure (general)*							
1-deoxy-D-xylulose 5-phosphate synthase	DXPS	MG066	*B. subtilis*	BSU24270	2E-09	2E-10	
Phosphoglucomutase	PGMT	MG053	*B. cereus*	BCE_5058	2E-67	2E-67	
glutamine-N5 methyltransferase	GLNMT	MG259	*E. coli*	b1212	1E-10	6E-11	

a Encodes the phosphocarrier protein. The phosphoenolpyruvate-protein phosphotransferase component (MG429) was already included in the model.

^b^ Forward referes to using the *M. genitalium* locus as the input sequence to be searched against other genomes.

^c^ Reverse referes to using the locus of the other organism as the input sequence to be searched against the genome of *M. genitalium*.

### Generation of Computations-Ready Model

A metabolic reconstruction has been described as a 2-D annotation of a genome [32]. The generation of a computations-ready model requires the complete assignment of metabolites to reactions, inclusion of exchange reactions, resolution of gene-enzyme associations, and derivation of biomass equations. Here, we largely follow the steps put forth by [33] in the latest *E. coli* metabolic reconstruction.

**Figure 3:**
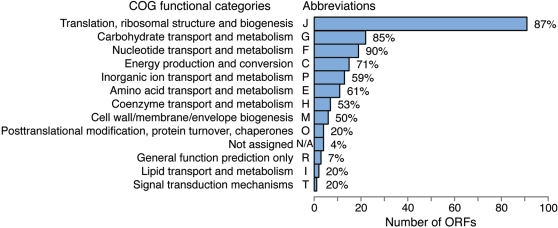
Classification of the ORFs included in *i*PS189 grouped into COG functional categories. The percent assigned to each class refers to the coverage of the total number in the genome accounted for in the model. Some of the ORFs in *Mycoplasma genitalium* do not currently have a COG functional category assignment (here represented as N/A). Note that although each ORF is only counted once within each COG functional category, some ORFs have multiple COG category assignments.

- *Assignment of ORFs, reactions & metabolites and their classification*. Under this step we first include in the model all reactions associated with enzymes as identified above (ensuring, when necessary, that the metabolites have the appropriate protonation state corresponding to pH 7.2 and are elementally balanced). We then extend the list to include non-gene associated reactions (e.g., account for the substrates and products for the kinase pool). Subsequently, we append spontaneous reactions for chemical transformations that are not enzyme catalyzed and for the transport of molecules that can freely diffuse across the cell membrane and are likely to be present in the natural or laboratory-adopted environment of *M. genitalium*. A distribution of the ORFs, metabolites, and reactions included in the final model *i*PS189 are provided (see Tables S1, S2, S3 and Text S1). A breakdown of the ORFs into functional clusters is shown in Figure 3. Here, each ORF is assigned to a cluster of orthologous groups (COGs) ontology [35] along with the percent of the total ORFs for the entire *M. genitalium* genome that is included in *i*PS189 for each classification.
- S*ystem boundary identification*. The system boundary includes the entire reaction network. Exchange reactions were created that allowed metabolites to enter or leave the extracellular space. Constraints on these exchange reactions were established to account for the simulated growth conditions by restricting the uptake (substrate) metabolites of the system.
- *Conversion of model into computations-ready form*. The stoichiometric matrix for *i*PS189 after step 1 involves 179 rows (i.e., reactions and exchange reactions) and 292 columns (i.e., total metabolites, taking the compartment designations into account). We also established gene-protein-reaction (GPR) associations to link reactions to genes. Only 48 reactions contained more than a single GPR association. Of these, none contained single-protein isozymes, and 47 were catalyzed by just one of the various enzyme complexes (among which was the ribosome and its associated proteins). Only one contained both isozymes and protein complexes.
- *Biomass equation derivation*. The derived biomass equation drains all metabolites present in cellular biomass in their appropriate molar biological ratios (see Table 3 for a list of components). Because the dry cell content of *M. genitalium* has not been experimentally measured, the biomass composition was estimated from the protein composition of closely related mycoplasmas, comparisons with a metabolic model for the related *Bacillus subtilis*, and a reduction of the biomass equation from the *Escherichia coli* model *i*AF1260. These biomass compositions were adjusted to account for components known to be absent in *M. genitalium* such as the cell wall. The biomass equation explicitly includes charged and uncharged tRNA molecules, rather than only the amino acids themselves. It also contains a non-growth-associated ATP maintenance reaction. The genome and RNA content was adjusted to reflect the low G+C content of *M. genitalium*. While determining the precise biomass composition has been a challenge for many other models [36], previous work has shown that the calculated biomass production is relatively insensitive to the exact ratios used [37].

**Table 3:** Biomass components used in *i*PS189.

Protein^a^		RNA	Inorganic Ions	Cofactors and Other
L-alanine	L-lysine	ATP	ammonium	FAD
L-arginine	L-methionine	GTP	calcium	NAD
L-asparagine	L-phenylalanine	CTP	chlorine	NADP
L-aspartate	L-proline	UTP	cobalt	coenzyme A
L-cysteine	L-serine		copper	10-formyltetrahydrofolate
L-glutamine	L-threonine	**DNA**	iron	5,10-methylenetetrahydrofolate
L-glutamate	L-tryptophan	dATP	magnesium	5,6,7,8-tetrahydrofolate
Glycine	L-tyrosine	dGTP	manganese	pyridoxal 5′-phosphate
L-histidine	L-valine	dCTP	molybdate	S-adenosyl-L-methionine
L-isoleucine	N-formylmethionine	dTTP	nickel	Spermidine
L-leucine			phosphorous	riboflavin
			potassium	putrescine
			sulfate	menaquinol 7
			zinc	mycolic acid
				minor teichoic acid^b^
				teichuronic acid^b^
				triglucosyl-1,2 diacylglycerol^b^

a Amino acids were incorporated as charged and unchanged tRNA molecules in the equation as reactants and products, respectively. The charged gln-tRNA becomes an uncharged glu-tRNA and the charged fmet-tRNA becomes an uncharged met-tRNA through the biomass equation.

b Added during GrowMatch procedure.

The computations-ready model along with the biomass description allows for the use of optimization-based techniques for testing and correcting for the presence of connectivity gaps (step 3) and growth prediction inconsistencies (step 4).

### Analysis and Restoration of Network Connectivity

The initial model was constructed almost exclusively based on homology searches within model libraries. This procedure led to the presence of many network gaps [17] preventing 177 reactions (99% of total) from carrying flux under all uptake conditions (i.e., they were blocked). As a consequence, these blocked reactions precluded the formation of some of the biomass components. Using GapFind [17] we found that a total of 175 (70%) cytoplasmic metabolites could not be produced inside or transported into the intracellular space. These metabolites included a number of biomass precursor metabolites (e.g., some amino acids, cofactors and metal ions) that had not been assigned uptake reactions. Of all the blocked metabolites, thirteen were involved in nucleotide metabolism and eight were metal ions without an identified transporter. We also note that 40 of these metabolites are charged/uncharged tRNA molecules, which are active in closed reaction cycles used in forming the protein component of biomass.

Through the use of GapFill [17] we subsequently sought to bridge these network gaps through the addition of reactions, transport pathways and relaxation of irreversibilities of reactions already in the model. Reactions known not to be present in *M. genitalium* (e.g., an incomplete TCA cycle) were excluded as gap filling candidates. We first applied GapFill to unblock constituents of biomass guided by the known components in the growth medium. We unblocked biomass production by adding 65 reactions, for which most (i.e., 43) were involved in metabolite transport, such as for the uptake of amino acids (14), folate, riboflavin, metal ions (8), and cofactors such as CoA. Among the remaining reactions were those responsible for the hydrolysis of dipeptides (15) and eight reactions involving other biotransformations. We performed an additional round of BLASTp comparisons of genes annotated with these reactions against the *M. genitalium* genome to determine if we could associate any of these reactions with specific genes in *M. genitalium*. We found five proteins catalyzing these reactions that had BLASTp scores smaller than 10^−5^ (see Table 2). For example, GapFill suggested the addition of reaction glutamyl-tRNA(Gln) amidotransferase in the model to allow the formation of the gln-tRNA molecule. BLASTp searches allowed us to link this activity with the genes encoding for the three subunits (MG098, MG099 and MG100). Note that these three genes (and others added during this step) were not added earlier (steps 1 and 2) on account of their ambiguous functional characterization. By bringing to bear both homology (though BLASTp) and connectivity restoration (through GapFill), here we rely on multiple pieces of evidence when appending a new functionality and corresponding genes to the model.

**Figure 4:**
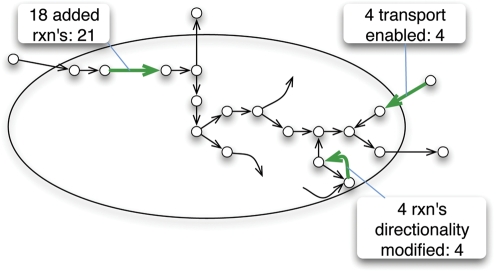
Summary of the GapFind and GapFill procedures after biomass unblocking. During biomass formation unblocking, most metabolites that could not be produced (disconnected metabolites) were reconnected through the addition of transport reactions. Metabolites not directly connected to biomass were mostly reconnected through the addition of reactions from KEGG and Metacyc.

Even after unblocking biomass formation, 43 metabolites remained blocked and were subsequently analyzed by GapFill. The results from GapFill are summarized in Figure 4. We were able to reconnect three metabolites by treating three reactions as reversible. We also found that the originally assigned (based on the auto-model) directionality of 1-acyl-sn-glycerol-3-phosphate acyltransferase was incorrect. It was subsequently reversed and found to be in accordance with both KEGG and MetaCyc entries. An additional 21 metabolites were reconnected by adding 18 reactions from the KEGG and MetaCyc databases (see Materials and Methods). The addition of these 18 reactions also introduced an additional nine metabolites (three of which were involved in glycerolipid metabolism) to the model. Finally the incorporation of uptake/transport reactions reconnected an additional four metabolites. We performed an additional round of BLASTp comparisons and we were able to associate three out of 22 reactions with specific genes (see Table 2). We found that the associated gene (*i.e.* MG066) for 1-deoxy-D-xylulose 5-phosphate synthase was already included in the model but with a different functionality (i.e., transketolase). The secondary synthase functionality, revealed by GapFill/BLASTp, was subsequently associated with gene MG066 in the model. A similar situation occurred with MG053, which was already associated with phosphomannomutase in the model. In addition, gene MG259 (annotated as “modification methylase, HemK family” in the Comprehensive Microbial Resource, http://cmr.jcvi.org) was added to the model to carry out the glutamine-N5 methyltransferase activity elucidated by GapFill/BLASTp. The model statistics after correcting for network gaps are summarized in Table 1.

### Model Correction Using *In Vivo* Gene Essentiality Data

Based on *in vivo* gene essentiality data [19] we deduced that there are 174 essential genes and 19 non-essential genes among the 193 genes provisionally present in the model (after steps 1, 2, and 3). We note that the *in vivo* gene essentiality experiments were performed using non-defined medium containing serum and yeast hydrolysate among other rich components. During the *in silico* model predictions/comparisons, we allowed the uptake of all extracellular metabolites with transport reactions, except for sugars other than glucose, in order to computationally approximate this medium.

Using a recently proposed diagnostic of the percentage of correctly-identified essential genes [38],[39], we found that the model correctly identified 137 out of a total of 174 essential genes (i.e., specificity of 79%) and 16 out of a total of 19 non-essential genes (i.e., sensitivity of 84%). This implies that the model (after steps 1, 2, and 3) was 79% correct in its overall accuracy in growth predictions (i.e., 153 of 193). Most of the mismatches (92%) were over-predictions of the metabolic capabilities (i.e., predicting growth when none is observed *in vivo*) instead of under-predictions (i.e., predicting no growth when growth is observed *in vivo*). We subsequently deployed the GrowMatch method (Satish Kumar and Maranas, submitted) to rectify as many as possible of the erroneous essentiality predictions by the model. GrowMatch functions by identifying the minimal number of model modifications required to restore consistency between growth predictions and gene essentiality experiments (see Materials and Methods).

Model under-predictions include mutants (MG410 and MG411), which encode the subunits for the phosphate transporter, preventing in both cases the uptake of phosphate. This implies that *M. genitalium* must have an additional uptake route of phosphates. Even though GrowMatch suggested a number of phosphate uptake alternatives to resolve this conflict and Glass and coworkers [19] had posited the activity of a putative phosphonate transporter (MG289, MG290, and MG291), we decided not to add them to the model as no direct evidence exists to ascertain their presence. For instance, the putative phosphonate transporter might be nonspecific thus also enabling uptake of phosphate. Alternatively, the unidentified phosphonate substrate might be catabolized to yield phosphate through a number of reactions. The other incorrect under-prediction involved MG138 (homologous to elongation factor 4 in *E. coli*), which had been associated with macromolecule formation during the automodel construction. We observed that deletion mutants of the homolog in *E. coli* (*lepA*) are viable [40]. Based on this information, we removed this gene and its erroneous association as an essential component of the biomass equation from the model.

Interestingly, three of the 37 erroneous over-predictions were corrected by adding three membrane components to the biomass equation (see Table 3). These components were not added during the initial model construction because it was not clear which (if any) of this class of metabolites were essential. An additional three erroneous predictions of non-essentiality were corrected by suppressing two reactions. One of these reactions, inosine kinase, was added during GapFill but not linked to an associated gene. Suppression of this reaction corrected two over-predictions but did not invalidate any correct model predictions, suggesting that the reaction activity is unlikely to be present *in vivo*, at least under the experimental conditions, and perhaps is not an activity encoded by *M. genitalium*. An additional six over-predictions involved two metal ion ABC transporters. GrowMatch identified each transporter to be essential when the other one was suppressed. We rejected co-regulation of the two transporters as a model restoration mechanism. Instead, we restored consistency for three of the six genes by assigning the cobalt uptake to the complex with a better homology to characterized cobalt transporters (MG179, MG180, MG181). The remaining three genes were removed from the model. An alternative interpretation of the GrowMatch results is that some other ion uptake reaction(s) are uniquely associated with these transporters and are thus responsible for the *in vivo* phenotype.

**Figure 5:**
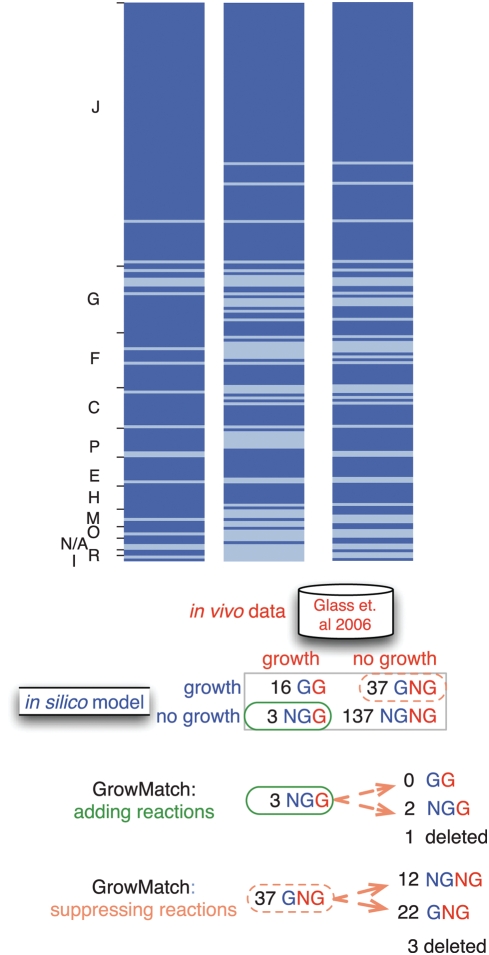
Summary of the GrowMatch reconciliations. The ORFs included in the comparisons are ordered within each COG functional category (see Figure 3 for abbreviations). If an ORF belongs to more than one category only the first one is used. The dark shading indicates an ORF that was essential, and the light shading indicates a non-essential ORF. Before the application of GrowMatch, 79% of the model predictions on gene essentiality agreed with *in vivo* experiments. Nearly all incorrect predictions occurred when the model predicted growth, but no growth was observed in the *in vivo* experiment (termed GNG mismatches). GrowMatch was able to rectify eighteen of these mismatches. This increased the percent agreement to 87%. The rightmost heat-map and the number of resolved mismatches also include the removal of one gene to correct a GNG experiment and three genes to resolve NGG mismatches.

Overall, the application of GrowMatch to the metabolic model led to the generation of a number of testable hypotheses regarding the presence or absence of specific functionalities and emphasized the importance of determining the substrate specificity of the transporters. We also identified reactions that had to be inactivated only for certain knock-outs suggesting their dependence on the genetic background in addition to the specific environmental conditions. For example, MG112 (ribulose-phosphate 3-epimerase) had to be suppressed in conjunction with two single gene deletions (i.e., conditional suppressions) to restore consistency with the *in vivo* data, suggesting possible regulation events. Figure 5 summarizes the complete GrowMatch results. Considering only those changes that could be repaired with global model adjustments conservatively raised the overall percent accuracy of the model (versioned as *i*PS189) from 79 to 87%. By recalculating the diagnostics of the percentage of correctly- identified essential genes [38],[39] we found that the model is now 87% (i.e., 149 of 171) correct in its essentiality predictions (specificity) and 89% (i.e., 16 of 18) correct in its non-essentiality predictions (sensitivity).

### *M. genitalium i*PS189 Model Characteristics

The *i*PS189 model predicts that *M. genitalium* uptakes fructose via a PTS system. The fructose is converted to fructose 1,6-bisphosphate (fdp) and finally enters the glycolytic pathways to produce lactate via lactate dehydrogenase. Neither fructose nor glycerol uptake was found to be essential as glucose could be efficiently taken up and converted. Specifically, glucose is transformed to glucose 6-phosphate and finally to fdp via phosphofructokinase. As expected, the model also indicates that co-enzyme A (CoA) is taken up, since *M. genitalium* has no coA biosynthesis genes. Additionally, accetal-CoA (accCoA) is not formed via pyruvate formate lyase but rather by pyruvate dehydrogenase. Interestingly, we find that should acetate be taken up, it is converted to acetyl phosphate (actp) and finally to accoa by phosphotransacetylase. Sources for acyl-CoA (aCoA) and CDP-glucose are also required for lipid production. The metabolites riboflavin and nicotinic acid (niacin) are taken up for synthesis of the cofactors FAD and NAD, respectively.

In addition, both spermidine and putrescene are directly imported as biomass components. Similarly, we also found that D-ribose (rib-D) is needed to fuel the truncated pentose phosphate pathway. Examination of fluxes indicated that the uptake of rib-D results in production of 5-phospho-α-d-ribose 1-diphosphate, which enables the conversion of adenine to amp. We also deduced that only adenine and cytidine are precursors to nucleotides and nucleosides (CTP, dCTP, UTP, dUTP, dTTP). Interestingly, the model required the direct uptake of GTP and could not be produced through the uptake of guanine. Model modifications that eliminate this requirement using GrowMatch resulted in a number of incorrect gene essentiality predictions. The need for the direct uptake of GTP is consistent with the fact that in *M. mycoides* the guanine nucleotide pathways depend on transport of preformed guanine derivatives [34], and that a number of other Mycoplasmas are not able to grow on medium that only contains guanine as a nucleobase [41]. In addition, all amino acids are imported directly from the environment as either monomers or dipeptides. Unlike many other mycoplasmas, *M. genitalium* is an arginine nonfermenting species, and not surprisingly arginine deiminase activity was not present in the model. Furthermore, in *i*PS189, the only participation of the amino acid arginine is its direct incorporation into biomass. Finally, flux predictions revealed that lactate is the main product of *M. genitalium* fermentation.

### Identifying Necessary Components of a Defined Medium

A key targeted application of the *i*PS189 metabolic model is to drive the development of a defined growth medium. As noted above, gene essentiality experiments were performed using a non-defined medium, SP-4, which contains beef heart infusion, peptone supplemented with yeast extract and fetal bovine serum. The use of an undefined medium can confound the characterization of gene essentiality, as the exact environmental conditions are not fully specified. Furthermore, the lack of a defined growth medium complicates the understanding of nutritional control and regulation of pathologies, evaluation of drug susceptibility, characterization of auxotrophic mutants and performing genetic analysis.

Using trial-and-error researchers have already attempted to formulate defined media by systematically deleting components from an undefined or complex media [28]. For example, defined media have been constructed for the growth of *Mycoplasma capricolum* [42], *Acholeplasma laidlawii* [43], *Spiroplasmas* [44], and a semi-defined medium was recently formulated for two *Mycoplasma mycoides* subspecies [45]. However such approaches do not take into account the balance and availability of chemical species in cellular metabolic pathways to systematically guide medium design. Genome-scale models of metabolism provide maps for tracing missing components needed for biomass formation, redox potential and ATP maintenance [46]–[48]. These models have already been successfully employed to establish minimal reaction sets needed for growth under several uptake environments [49], elucidate substrate uptake requirements for several microbial organisms such as *Helicobacter pylori* [50] and *Haemophilus influenzae* [50] and more recently design complete growth media [51]–[53].

Motivated by these medium-associated shortcomings, we used the *i*PS189 metabolic model as a roadmap of the available transporters, metabolites and internal interconversions to seek out the minimum number of growth medium components necessary for biomass production. We used as a starting point the components of the C5 medium (Rodwell, 1983), which is used as a component of SP-4, with the addition of folate and biotin and the replacement of thiamine by the four individual deoxynucleosides (see Table S4). By minimizing the total number of additional components that are needed for growth (see Materials and Methods), a number of additional components were identified as required (see Table 4). The purported reduced bio-availability of the amino acids in their monomeric form might be, in part, the reason for *M. genitalium*'s requirement of yeast extract. The listed dipeptides were found to be needed components consistent with the presence of dipeptide transporters that were found to be essential genes both *in vivo* and in *i*PS189. In addition to components identified based on the metabolic model, we identified that some of the enzymes present in the model required cofactors not included present in the model. In addition, a number of additional carbon source supplements could be investigated.

**Table 4:** Metabolites computationally predicted as necessary supplements to the modified C5 medium in order to support growth of *M. genitalium*.

*Essential cofactors and components without a synthesis pathway*		
5-amino-4-oxopentanoate	menaquinone	pyridoxal
Arbutin	2-oxoglutarate	salicin
Cytidine	6-phospho-D-gluconate	thiamin
D-glucosamine	putrescine	thymidine
GTP		
*Metal ions*		
Ca	Fe	Cu
Zn	Co	Mn
Mo	Ni	
*Dipeptides*		
ala-L-asp-L	gly-glu-L	gly-met-L
met-L-ala-L	gly-asp-L	gly-pro-L
cys-gly	ala-L-gln-L	ala-L-glu-L
ala-L-gly	ala-L-his-L	ala-L-leu-L
ala-L-thr-L	gly-asn-L	gly-gln-L
*Supplemental carbon source*		
fructose^a^		
*Component ‘replaced’ in modified C5 but present in C5*		
thymine^a^		

a This component was not predicted as essential for growth, but its inclusion improved biomass yield.

We note that these medium predictions generate a necessary but not a sufficient list of components needed in the medium. For instance, a number of components of biomass such the lipids are not fully specified in the model. Furthermore, additional signaling molecules might be necessary signals for allowing growth of *M. genitalium* in a defined medium. We anticipate that the iterative process of testing and refining a defined medium followed by updates to the model will successively help pinpoint the precise metabolic capabilities and requirements of *M. genitalium*.

## Discussion

In this paper, our focus was two-fold: (a) to construct the metabolic model for the minimal organism and pathogen *M. genitalium*, and (b) to introduce and bring to bear automated procedures that streamline the construction of metabolic models. The procedure does not require a fully annotated genome and can serve to complement existing annotations [2] by generating testable hypotheses of functionality. We made use of BLASTp to associate functionality to ORFs in the multiple stages, but alternate methods of determining enzymatic function, such as profile-based approaches [54],[55] could provide additional or alternative assignments. Hypothesizing novel pathways [56] will also likely become increasingly important as metabolic reconstructions for more diverse organisms are carried out.

Many genome annotation errors are caused [57] by the use of non-specific reaction compound associations or partially qualified Enzyme Commission numbers [58]. For instance, a comparative study identified an 8% difference in ORFs across three different functional annotations of *M. genitalium* [59]. Therefore, the direct use of genome-annotation derived reconstructions (e.g., KEGG and Pathway Tools generated models) can lead to inaccurate descriptions of metabolic behavior [60]. Specifically, a recent study has shown that a permissive inclusion of pathways from these reconstructions can lead to models that overpredict the metabolic capabilities of *Lactococcus lactis* [61]. To safeguard against this issue, we have used manually curated metabolic models as libraries of biotransformations. We note that this procedure allows for the straightforward incorporation of reactions that are charge and elementally balanced, which is not the case with many reactions in KEGG [62]. Earlier efforts have examined the general metabolism of Mycoplasmas [63] or targeted some specific subsections such as purine and pyrimidine metabolism [41],[64]. Although there is an overlap between the reaction set in our reconstruction and those available in previous studies, developing a *M. genitalium* specific model with growth requirement as a constraint revealed novel uptake and non-gene associated reactions that previous studies were unable to identify.

The identification of which metabolites are produced internally or transported directly from the extracellular environment was complicated by the lack of a defined medium for *M. genitalium* and its fastidious growth-requirements. Notably, no other metabolic model reconstruction efforts to date were complicated by the lack of both a well-defined biomass composition and a defined growth medium. Here, we allowed the uptake of all metabolites known to be present in the current medium. We also included metabolites either with identified transporters or those necessary for reconnecting blocked metabolites/reactions. Even though missing metabolites and pathways still exist in *i*PS189, we were able to achieve a high degree of agreement between the model predictions and *in vivo* gene essentiality data (87%). We note that the most recent iteration of the metabolic model for *E. coli*, an organism which has both a well-defined biomass composition as well as chemically defined growth media, has an overall agreement with *in vivo* gene essentiality data of 91% under aerobic glucose conditions [33]. Becker and Palsson [38] have recently reported that most *in silico* models correctly predict less than 45% of essential genes (called specificity in [39]); for the most-recent *E. coli* model this diagnostic was 66%. *i*PS189 has similar performance on both the overall accuracy in growth predictions and specificity (both 87%) because of, in part, the much higher percentage of essential genes in *M. genitalium* but also its careful construction. Additional *in vivo* gene essentiality studies using a fully defined medium could usher a more accurate elucidation of the true metabolic capabilities of *M. genitalium*, as well as suggest improvements to the reconstruction.

The metabolic model *i*PS189 is smaller than the 256 genes suggested as a minimal gene set based on comparison of *M. genitalium* and *H. influenzae* proteins [22]. In large part, this seeming discrepancy results from the intentional exclusion from the model of many genes that are essential (e.g., those encoding DNA and RNA polymerases) though not directly related to metabolic processes. If such genes were included, the model size would increase by (at least) 59 genes to 248. In addition, 68 genes that are essential *in vivo* have unknown function, and thus cannot (yet) be incorporated into the model. It is possible that they could, in part, carry out some of the non-gene associated reactions that were proposed during the GapFill procedure. Determining their metabolic function through biochemical and molecular biology techniques, as well as determining the substrate specificity for non-characterized transporters, would improve subsequent metabolic models.

Looking to the future, we note that it was recently shown that it is feasible to transplant the genome from one mycoplasma species to another [65], thus opening the door to the transplantation of a perhaps completely synthetic genome. Furthermore, the recent announcement of the *de novo* synthesis and assembly of the complete *M. genitalium* genome [66] brings closer to reality the *ab initio* design of microbes from scratch that are exquisitely tuned for specific biotechnological applications. The constructed metabolic model *i*PS189 could serve as a core of metabolic functions to add upon so as to bring about the desired biological functionalities and/or production capabilities.

## Materials and Methods

The general principles of the metabolic reconstruction process have been previously outlined [1],[51]. In the following section, the specific methods used in the reconstruction *of M. genitalium* are detailed.

### Initial Reconstruction Content

The first step in our reconstruction of the genome-scale model of metabolism of *M. genitalium* involved analyzing the annotated *M. genitalium* G-37 genome sequence with the SimPheny automated model generation platform developed by Genomatica (San Diego, CA). This automated procedure leveraged the content contained in Genomatica's manually curated models to expedite the initial reconstruction of *M. genitalium* metabolism. For the *M. genitalium* model, reconstructions for *E. coli*, *Haemophilus influenzae*, *Geobacter sulfurreducens*, *B. subtilis*, and *Saccharomyces cerevisiae* were used in the comparisons. Subsequently genes-protein-reaction (GPR) associations were established based on the evidence provided by homology identity provided by sequence analysis and annotation information. Specifically, forward and reverse protein-protein BLAST (BLASTp) comparisons were carried out to identify genes in the library of manually curated models that most closely resemble those in *M. genitalium*. In the forward BLASTp step, each *M. genitalium* ORF was compared against each gene in the manually curated models. Then, in the reverse BLASTp step, the best hit from manually curated models was compared against the *M. genitalium* genome ORFs. The top hits for each of the searches were stored, and a third list was compiled consisting of the cases in which the top hits are identical in both the forward and reverse BLASTp searches. For only these pairs, the GPR association(s) from the manually curated model was used as a template to assign those in the *M. genitalium* model. This two-way BLASTp search procedure improves the likelihood that *M. genitalium* orthologs, not paralogs, to genes in the manually curated models are identified and assigned by the automodel process.

Although the automated genome-model comparisons enabled a fast generation of a draft model, the auto-generated reconstruction was extended by searches for relevant reactions not included in the library of curated models. First, all high-quality annotated “non-metabolic” genes, such as those encoding transcription factors and proteins involved in replication and repair, were excluded from subsequent searches. We then extracted the remaining sequences of all the ORFs not yet included in the *M. genitalium* model and performed BLASTp analysis to identify additional proteins with *biochemically characterized* functionalities in other organisms. The target database was the nr (non-redundant) database at NCBI (http://www.ncbi.nlm.nih.gov/blast). Next, all target proteins that the BLASTp search reported Expect (E) values less than 10^−5^ were further investigated for biochemical characterization. Next, we carried out a reverse BLASTp analysis of each of these characterized proteins against sequences in the *M. genitalium* genome. If the initial *M. genitalium* gene was ranked with the best score and had an acceptable E value (less than 10^−5^) at the end of this step, that gene and its associated reactions were included in the model as with the library of curated models.

During the construction of the initial model, all of the reactions added during the auto-generated reconstruction were also subsequently examined for internal consistency. For example, reactions involving compartments (which are not present in the bacterium *M. genitalium)* were updated to occur in the cytosol, three reactions for ATP synthase were merged, and two macromolecule synthesis reactions were combined and their GPR associations were manually updated. We also added GPR associations for missed characterized members of complexes (e.g., a component of an ABC transporter system) when the automodel associated the other members, as well as corrected those GPR associations for proteins that were misidentified as isozymes instead of complexes. Furthermore, we investigated the cluster of orthologous groups (COGs) ontology [35] of all genes in the automodel, as well as examined all ORFs using the NCBI nr database as an added guard against the erroneous transfer of a functionality into the *M. genitalium* model. Those having classification within non-metabolic classifications, such as transcription (K); replication, recombination and repair (L); general function prediction only (R); function unknown (S); or none assigned (N/A) were inspected further. Although most of the ORFs within (R) and (N/A) were retained, we removed all those within (K), (L), and (S) from the model (see Table S3). All reactions, including those added from the BLASTp searches and subsequent additions, were checked and corrected whenever necessary for charge balance (protonation state) and elemental balance.

### Generation of the Biomass Equation

The biomass equation was generated by accounting for as many as possible of the constituents that form the cellular biomass of *M. genitalium*. Unfortunately, no complete compilation is available in the open literature for *M. genitalium*, however some information on the soluble protein composition of it [67] and data on closely related mycoplasmas is available. Therefore, we started with the biomass equation from the Gram positive *B. subtilis* model [68] and the core biomass equation from the *E. coli i*AF1260 metabolic model [33] and subsequently modified them accordingly. First, all components related to the cell wall were removed as *M. genitalium* lacks one. Next, the amount of precursor molecules involved in DNA production were adjusted by altering their ratios in accordance with the lower G+C content in *M. genitalium* (31.7%) as compared to *E. coli* (49.8%) and *B. subtilis* (40.9%) and its smaller genome size. The amino acid relative percentages were likewise adjusted based on utilization patterns and codon biases. Amino acid utilization was incorporated as charged and uncharged tRNA molecules in the biomass equation as reactants and products, respectively. Note that the charged gln-tRNA becomes an uncharged glu-tRNA and the charged fmet-tRNA becomes an uncharged met-tRNA through the biomass equation. Metal ions known to be present in the active sites of catalytic enzymes were also included in the biomass equation. Various membrane and lipid components were subsequently added in accordance with the GrowMatch predictions.

### Generation of Computations-Ready Model

Testing the metabolic model using optimization-based approaches requires the definition of a number of sets and parameters.

Sets:
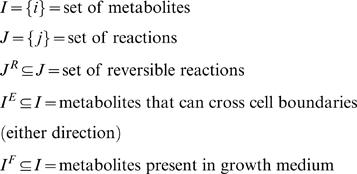

Parameters:
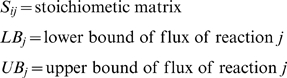

Variables:
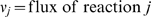

Upper and lower bounds, *UB_j_* and *LB_j_*, were chosen as not to exclude any physiologically relevant metabolic flux values. The upper bound for all reactions was set to 1,000. The lower bound was set equal to zero for irreversible reactions and to −1,000 for reversible reactions. The non-growth associated ATP maintenance limit was set to *LB_j_* = 8.4 gDW^−1^ h^−1^. The maximum transport rate into the cell was 5 mmol gDW^−1^ h^−1^ for any external carbon containing metabolite (*i.e.*, *LB_j_* = −5). The lower bound for the remaining source exchange reactions was −20 mmol gDW^−1^ h^−1^ [33].

Using the principle of stoichiometric analysis along with the application of a pseudo-steady-state hypothesis to the intracellular metabolites [69], an overall flux balance can be written as follows:
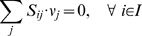
(1)


When constructing the model, we also generated the gene-protein-reaction (GPR) associations that link the ORFs to the reactions that are catalyzed by their gene products using standard conventions [70].

### Analysis and Restoration of Network Connectivity

Once a mathematical representation of the metabolic model was generated, we first determined using GapFind [17] which metabolites could not be produced (*i.e.*, cannot carry any net influx), given the availability of all substrates supported by the model. We next applied GapFill to modify the existing genome-scale model in order to reconnect these metabolites to the model. The reaction source databases used by GapFill in this work were the KEGG [71] and MetaCyc [72] databases. These reactions were provisionally added to the model, after evaluating charge and elemental balancing. We also performed additional homology searches to try to identify any additional GPR associations. Care was taken when applying GapFill so as not to introduce functionalities known to be absent in *M. genitalium* (e.g., incomplete TCA cycle and disconnected pentose-phosphate pathway).

### Model Correction Using In Vivo Gene Essentiality Data

The introduction of the GPR associations introduces additional complexity in that an additional layer of detail is needed to fully characterize the network when a single gene is deleted. We made the following definitions to this end:

Sets:
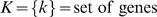

Parameters:
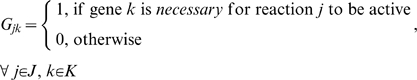

Variables:
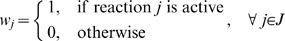

Set 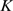 contains all ORFs (genes) that are included in the metabolic reconstruction. We used a fictitious gene *s0001*, as in [33], to map and track spontaneous reactions that are known to be non-enzymatic (e.g., diffusion of CO_2_ across the cell boundaries), but this fictitious gene was not included when enumerating the total genes included in the model, nor could it be knocked out. Note that a gene *k* may have more than one GPR association when it is involved with more than one reaction. Likewise, a reaction *j* may be involved in more than one GPR association, as is the case with isozymes or multi-protein complexes. We restricted reaction fluxes, *v_j_*, using the binary variable *w_j_* as follows:
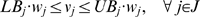
(2)Equation (2) ensures that the flux in reaction *j* can take a non-zero value only if the reaction is active (i.e., *w_j_* = 1).

Parameter 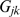 describes the impact of the deletion of gene *k* on reaction *j*. For instance, if only a single gene *k_1_* is associated with reaction *j*, then we assign 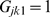. On the other hand, if two genes *k_1_* and *k_2_* encode isozymes that catalyze reaction *j*, then we assign 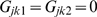. However, if two genes *k_1_* and *k_2_* are required for the formation of a multi-protein complex that catalyzes reaction *j*, we set both 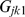 and 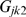 equal to one. More complex associations were handled in a similar manner.

We tested the *in silico* growth predictions of the *M. genitalium* metabolism network by examining the flux of the biomass equation. Given the deletion of a single gene *k*, we solved the following formulation:
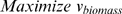
(3)subject to
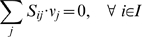
(1)

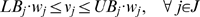
(2)

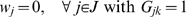
(4)Equation (4) ensures that the flux in reaction *j* is zero when the gene *k* that is necessary for its activity is deleted. Note that this equation takes advantage of the fact that in this work we only examined single gene deletions and thus needed not write more complicated GPR-related constraints. This formulation was solved for each gene *j* in the model using CPLEX version 11 accessed within the GAMS modeling environment. The predictions were compared against *in vivo* gene essentiality data [19]. Because the experiments were performed in a non-defined rich medium [19], all metabolites that had identified transporters or were known to be able to cross the membrane into the cell in a non-mediated way were allowed to have exchange reactions to enter the system. Exceptions were the metabolites known not to be present in the growth medium: sugars other than glucose, as well as acetate and lactate. In this study, we used the growth cutoff was that proposed in the recent study [73] which is defined as one–third of the *average* growth exhibited by all the single gene deletions under consideration. However, we found that the *in silico* growth predictions for *i*PS189 were insensitive to this value.

We next applied the GrowMatch method to reconcile inconsistencies between *in silico* and *in vivo* growth predictions across single gene deletion mutants (Satish Kumar and Maranas, in preparation). To this end, we first classified growth prediction inconsistencies into two categories: (a) a mutant is termed a “Grow/NoGrow” (GNG) mutant if the *in silico* model predicts growth whereas there is no observed growth *in vivo* and (b) a mutant is termed a “No Grow/Grow” (NGG) mutant if the *in silico* model predicts no growth in contrast with observed *in vivo* growth. In GNG mutants, the model overpredicts the metabolic capabilities of the organism. GrowMatch automatically restores consistency in these mutants by suppressing reaction activities to prevent *in silico* growth (i.e., by identifying erroneously added reactions or missing regulation). Conversely, in NGG mutants, the model underpredicts the metabolic capabilities of the organism. GrowMatch restores consistency in these mutants by adding functionalities that ensure *in silico* growth consistent with *in vivo* data. As when GapFill was applied, the additional reactions were carefully monitored. In all cases, GrowMatch operates so as not to perturb any correct growth predictions.

### Model-Guided Identification of Necessary Medium Components

We pose the problem of identifying the minimum number of added components to the growth medium so as to allow for the formation of all biomass constituents as an optimization problem. To this end, we introduce the following additional sets and variables:

Sets:
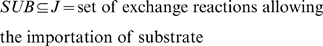

Variables:
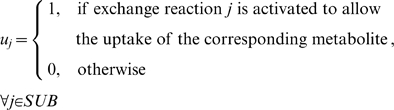

We minimized the total number of activated substrate exchange reactions that enable the uptake of growth medium components through the use of the following optimization formulation:
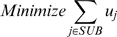
(5)subject to
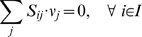
(1)

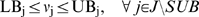
(6)

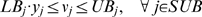
(7)

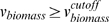
(8)Equation (6) ensures that the fluxes for all reactions that are not associated with the uptake of a substrate are within the bounds defined earlier. When an exchange reaction *j* is active (*u_j_* = 1), Equation (7) permits its flux to assume non-zero negative values, thus allowing the uptake of the corresponding substrate. Conversely, when an exchange reaction *j* is inactive (*u_j_* = 0), Equation (7) ensures that the exchange reaction can only remove the corresponding product from the extracellular environment (i.e., its flux can only assume positive values). Constraint (8) ensures a minimum amount of biomass formation, with the cutoff set to be the same as that used for the gene essentiality predictions above (i.e., one–third of the average maximum biomass flux exhibited by all the single gene deletions). This MILP problem was also solved using CPLEX version 11 accessed within the GAMS modeling environment.

## Supporting Information

Table S1 Reconstruction metabolite and reaction content
(0.15 MB XLS)

Table S2 Composition of the biomass equation
(0.03 MB XLS)

Table S3 Gene content of the model during each step of the reconstruction
(0.05 MB XLS)

Table S4 Components included in modified C5 medium
(0.02 MB XLS)

Text S1 *i*PS189 in SBML format
(0.02 MB ZIP)
